# Circulating micrornas as potential diagnostic biomarkers for cervical intraepithelial neoplasia and cervical cancer: a systematic review and meta-analysis

**DOI:** 10.1007/s12672-024-01028-7

**Published:** 2024-05-27

**Authors:** Yue Li, Longbiao Zhu, Chenjing Zhu, Yan Chen, Hui Yu, Hangju Zhu, Ping Yin, Mengyu Liu, Yang Li, Huixin Li, Zhen Gong, Hanzi Xu, Jing Han

**Affiliations:** 1 Jiangsu Cancer Centre, Jiangsu Cancer Hospital, Jiangsu Institute of Cancer Research, The AffiliatedCancer Hospital of Nanjing Medical University, Nanjing, Jiangsu China; 2 Department of The Sixth Dental Division, Affiliated Stomatological Hospital of Nanjing Medical University, Nanjing, Jiangsu China; 3 State Key Laboratory Cultivation Base of Research, Prevention and Treatment for Oral Diseases, Nanjing Medical University, Nanjing, Jiangsu China; 4 Jiangsu Province Engineering Research Center of Stomatological Translational Medicine, Nanjing, Jiangsu China; 5 Department of Gynecology, Women’s Hospital of Nanjing Medical University, Nanjing Woman and Children’s HealthCare Hospital, Nanjing, Jiangsu China

**Keywords:** Cervical cancer, Cervical intraepithelial neoplasia, Circulating miRNAs, Biomarkers, Meta-analysis

## Abstract

Cervical cancer is a prevalent malignancy of the female reproductive system. Cervical intraepithelial neoplasia (CIN) is a precursor lesion for CC. Various studies have examined circulating microRNAs (miRNAs) as potential early diagnostic markers for CC and CIN. However, the findings have been inconclusive. Therefore, it is necessary to evaluate the diagnostic accuracy and identify potential sources of variability among these studies. Methods: The PubMed, Cochrane Library, Embase, and Web of Science databases were searched to identify relevant literature. Then, Stata 14.0 was utilized to calculate summary estimates for diagnostic parameters, including sensitivity, specificity, positive likelihood ratio (PLR), negative likelihood ratio (NLR), diagnostic odds ratio (DOR), and area under the summary receiver operating characteristic (ROC). To scrutinize the heterogeneity, the Cochran-Q test and I^2^ statistic were utilized. As significant heterogeneity was observed, the random effects model was chosen. To explore potential sources of the heterogeneity, subgroup and regression analyses were conducted. Results: We analysed 12 articles reporting on 24 studies involving 1817 patients and 1731 healthy controls. The pooled sensitivity was 0.77 (95% CI 0.73–0.81), the specificity was 0.81 (95% CI 0.73–0.86), the PLR was 3.99 (95% CI 2.81–5.65), the NLR was 0.28 (95% CI 0.23–0.35), the DOR was 14.18 (95% CI 8.47–23.73), and the area under the curve (AUC) was 0.85 (95% CI 0.81–0.87). Subgroup analysis revealed that multiple miRNAs can improve diagnostic performance; the pooled sensitivity of multiple miRNAs was 0.78 (95% CI 0.68–0.86), the specificity was 0.85 (95% CI 0.78–0.90), and the AUC was 0.89 (95% CI 0.86–0.91). Conclusion: This study suggested that circulating microRNAs may be biomarkers for early CC diagnosis.

**Supplementary Information:**

The online version contains supplementary material available at 10.1007/s12672-024-01028-7.

## Introduction

Cervical cancer ranked fourth among the leading causes of cancer-related deaths in women worldwide in 2020, with approximately 604,127 new cases and 341,831 deaths [1].

Cervical intraepithelial neoplasia (CIN) is an intermediate stage preceding the onset of cervical cancer. Timely detection and treatment of CIN or early-stage cervical cancer yield more favourable clinical outcomes than treatment of advanced-stage patients. However, with a deficiency in early diagnostic biomarkers, the majority of cervical cancer (CC) cases are identified at later stages [2]. Therefore, prompt recognition of CIN and early-stage CC remains paramount. In addition, current cervical examination techniques, such as HPV DNA testing [3], Papanicolaou (Pap) smear [4], liquid-based cytology (LBC) [5], and colposcopy, are invasive. Hence, there is an immediate need for noninvasive biomarkers to detect CIN and CC.

MicroRNAs (miRNAs), which are approximately 22 nucleotides long, are intrinsic noncoding regulatory RNAs. miRNAs play a pivotal role in modulating physiological and pathological mechanisms by inhibiting or degrading target genes [6]. Recent research has revealed that circulating miRNAs can act as potent biomarkers for various types of cancer, including breast cancer [7], pancreatic cancer [8], non-small cell lung cancer [9], and cervical cancer [10].

Despite an increasing amount of research on the use of circulating miRNAs as diagnostic markers for CIN and CC, recent findings have been inconsistent, and a clinically viable panel for CC diagnosis is lacking. To address these shortcomings, we performed a comprehensive meta-analysis to evaluate the potential diagnostic significance of miRNAs in CIN and CC patients, comparing the diagnostic value of individual miRNAs versus multiple miRNAs.

## Materials and method

The methodology for this meta-analysis has been registered in the INPLASY.COM, an international database for prospectively registered systematic reviews (INPLASY202340053). The current meta-analysis follows the PRISMA-DTA statement of preferred reporting items for systematic reviews and meta-analyses of diagnostic test accuracy [11]**.**

### Search strategy

Publications were searched in four databases, including PubMed, Embase, Cochrane Library and Web of Science, without language estrictions until November 6, 2022. Utilizing the PICOS framework, our strategic sweep incorporated pertinent Medical Subject Headings (MeSH) and keywords procured from the National Center for Biotechnology Information (NCBI) platform. The detailed search strategy and keywords used are available in Supplementary Material: 1. Moreover, a manual search of relevant articles was also conducted to ensure the thoroughness of the search process.

### Eligibility criteria

The selection criteria mandated: (1) studies pertinent to the diagnostic effectiveness of circulating miRNAs in discerning CIN or CC; (2) inclusion of patients in the case group, diagnosed as per clinically approved criteria; and (3) obtainability of false positive (FP), true positive (TP), false negative (FN), and true negative (TN) frequencies, directly or indirectly. Conversely, any research falling under the exclusionary categories was excluded: (1) cellular, animal, or microbiological experiments; (2) non-comparative studies; and (3) reviews, meta-analyses, or conference summaries.

### Data extraction and quality assessment

Two independent reviewers extracted vital data from articles, covering author details, publication year, ethnicity, miRNAs profiles and expression levels, references, comparison type, sample size of all groups, detection methods of miRNAs, specimen type, AUC with 95% confidence intervals (CIs), and diagnostic performance data (sensitivity, specificity, TP, FP, TN, FN).

Bias risks were appraised utilizing the Quality Assessment of Diagnostic Accuracy Studies-2 (QUADAS-2) tool [12]. This instrument encompasses four key areas: patient selection, index test, reference standard, and flow and timing. A third reviewer was involved in when the consensus was not be reached.

### Statistical analysis

For calculating TP, FP, FN, and TN values, we obtained sample size, sensitivity, and specificity from each individual study. Statistical analysis was performed using Stata 14.0 software, including pooled sensitivity, specificity, positive and negative likelihood ratios, diagnostic ratios, and their respective 95% confidence intervals (CIs). Summary receiver operating characteristic (SROC) curves were also plotted to assess the area under the curve. Meta-DiSc 1.4 software was used to examine the threshold effect by evaluating Spearman's correlation coefficient and P value [13]. We appraised the heterogeneity among studies using the Cochran-Q test and I^2^ statistic for quantitative evaluation. Significant heterogeneity was inferred from a P-value below 0.05 for the Cochran-Q test or an I^2^ value exceeding 50%, prompting the application of a random-effect model in our analysis. Subgroup analyses and regression analysis served in identifying primary causes of heterogeneity. A sensitivity analysis was conducted to ascertain the reliability and robustness of the meta-analysis outcomes. We utilized Deeks’ funnel plots to check for potential publication bias. Fagan’s nomogram was devised for a more thorough assessment of miRNAs’ diagnostic effectiveness. Review Manager 5.4 assisted in evaluating the literature's quality, and a P-value less than 0.05 was adopted to substantiate the outcomes.

## Results

### Literature search and study characteristics

**Fig. 1:**
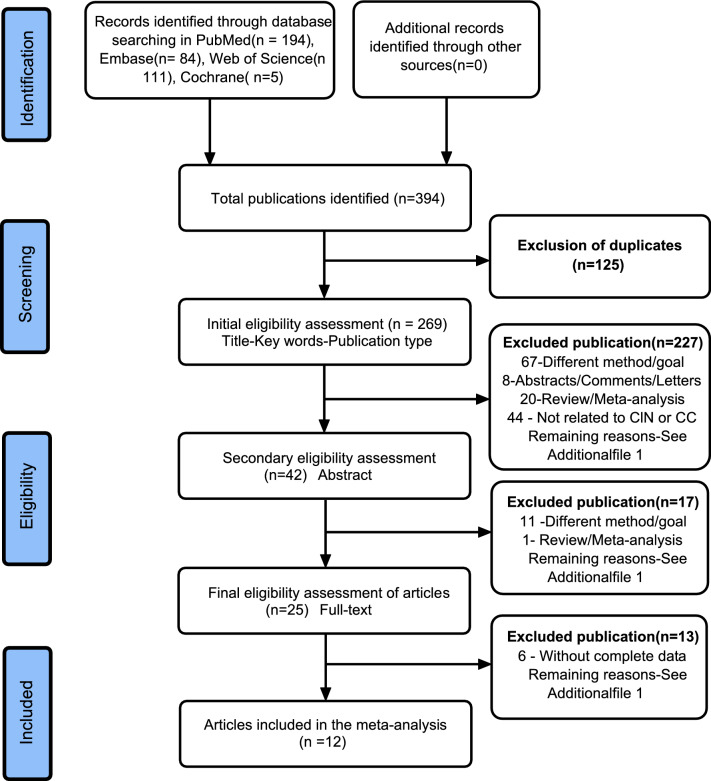
Flow diagram of screening the eligible studies

The search of four databases yielded an initial pool of 394 publications. After removing 125 duplicates and excluding 227 irrelevant papers based on the title, study type, and keywords, 42 potential articles remained. Subsequent assessment of the abstracts resulted in further exclusion of 17 papers, leaving 25 full-text papers for further review. Finally, 13 articles were excluded; therefore, 12 eligible articles published between 2014 and 2021 were included in this meta-analysis (Fig. 1 and Table 1).

**Table 1:** Summary of the exclusion reasons for all eligibility evaluation steps

Reason for exclusion	Number
Abstracts/Comments/Letters	8
Different method/goal	78
Different sample type	23
Metastatic focus	4
Not found	1
Not related to CIN or CC	44
Prognostic	9
Review/Meta-analysis	21
Therapeutic	63
Without complete data	6

The 12 included papers comprised 24 case‒control studies. The analysis involved 624 patients with CIN, 1193 patients with CC, and 1731 healthy controls. All of the included studies were published in English, and their primary findings are summarized in Table 2.

**Table 2:** The characteristics of the studies included in the meta-analysis

First author/year	miRNAs	Expression	Reference	Comparison type	Case/control	Method	Specimen	Sen(%)	Spe(%)	AUC (95% CI)
Cao,SP, 2021	miRNA-375-3p	Down	U6 snRNA	CC vs HC	124/119	qRT-PCR	Serum	75.80	86.6	0.723 0.869
Du,SY, 2020	miRNA-29a miRNA-25 miRNA-486-5p	Up	U6, miRNA-16, miRNA-25	CC vs HC	140/140	qRT-PCR	Serum	87.10	89.30	NA
Farzanehpour,M, 2019	miR-9	Up	U6 snRNA	CC vs HC	18/36	qRT-PCR	Serum	100.00	94.40	0.990 (0.990–1)
Farzanehpour,M, 2019	miR-192	Up	U6 snRNA	CC vs HC	18/36	qRT-PCR	Serum	100.00	94.40	1(1–1)
Farzanehpour,M, 2019	miR-205	Up	U6 snRNA	CC vs HC	18/36	qRT-PCR	Serum	88.20	88.90	0.960 (0.890–1)
Farzanehpour,M, 2019	miR-9	Up	U6 snRNA	CIN vs HC	18/36	qRT-PCR	Serum	77.80	94.40	0.900 (0.800–1)
Farzanehpour,M, 2019	miR-192	Up	U6 snRNA	CIN vs HC	18/36	qRT-PCR	Serum	83.30	94.40	0.980 (0.950–1)
Farzanehpour,M, 2019	miR-205	Up	U6 snRNA	CIN vs HC	18/36	qRT-PCR	Serum	66.70	88.90	0.750 (0.560–0.950)
Jia,WH, 2015	mir-21, mir-29A, mir-25, mir-200A, mir-486-5P	Up	Misture of let-7i, let-7 g and let-7d	CC vs HC	123/94	qRT-PCR	Serum	88.60	81.00	0.908 (0.868–0.948)
Li,J, 2014	P-miR-2	Down	U6 snRNA	CC vs HC	112/85	qRT-PCR	Serum	85.70	88.20	0.827 (0.767–0.887)
Kong,QQ, 2017	hsa-miR-92a	Up	Cel-39-3p	CC + CIN vs HC	86/40	qRT-PCR	Serum	69.60	80.40	0.830
Lv,AX, 2021	mir-125a—5p	Down	hsa-miR-16-5p	CC vs HC	44/28	qRT-PCR	Exosomal	59.10	84.00	0.713 (0.561–0.865)
Ning,RQ, 2021	HSA-miR-26B-5P HSA-miR-146B-5P HSA-miR-191-5P HSA-miR-484 HSA-miR-574-3P HSA-miR-625-3P	Up	hsa-miR-16	CC + CIN vs HC	240/211	qRT-PCR	Plasma	73.00	71.00	0.721 (0.711–0.731)
Ruan,F, 2020	microRNA-21	Up	U6 snRNA	CC vs HC	68/57	qRT-PCR	Serum	91.23	58.82	0.723 (0.631–0.815)
Ruan,F, 2020	microRNA-124	Down	U6 snRNA	CC vs HC	68/57	qRT-PCR	Serum	57.89	94.12	0.766 (0.677–0.856)
You,WZ, 2015	miR-127	Up	RUN6B	CC vs HC	68/49	qRT-PCR	Plasma	75.51	83.32	0.820 (0.739–0.903)
You,WZ, 2015	miR-205	Up	RUN6B	CC vs HC	68/49	qRT-PCR	Plasma	72.00	82.35	0.843 (0.767–0.920)
Zhang,YJ, 2015	miR-16–2* miR-195 miR-2861 miR-497	miR-16–2*:up miR-195:down miR-2861:down miR-497:up	Cel-miR-67	CC vs HC	184/193	qRT-PCR	Serum	73.10	88.40	0.849 (0.813–0.886)
Zhang,YJ, 2015	miR-16–2* miR-195 miR-2861 miR-497	miR-2861:down	Cel-miR-67	CIN vs HC	184/193	qRT-PCR	Serum	62.60	88.90	0.734 (0.683–0.784)
Zheng,SL, 2020	miR-638	Down	CEL-miR-39-3P	CIN vs HC	40/40	qRT-PCR	Serum	80.00	60.98	0.734
Zheng,SL, 2020	miR-521	Down	CEL-miR-39-3P	CIN vs HC	40/40	qRT-PCR	Serum	80.00	65.85	0.742
Zheng,SL, 2020	miR-203a-3p	Down	CEL-miR-39-3P	CIN vs HC	40/40	qRT-PCR	Serum	75.00	53.66	0.660
Zheng,SL, 2020	miR-1914-5P	Down	CEL-miR-39-3P	CIN vs HC	40/40	qRT-PCR	Serum	85.00	43.90	0.626
Zheng,SL, 2020	miR-296-5p	Down	CEL-miR-39-3P	CIN vs HC	40/40	qRT-PCR	Serum	71.79	58.54	0.623

Of the 24 studies, 19 focused on a single miRNA, while 5 focused on multiple miRNAs. Serum was the preferred medium for detecting 20 different miRNAs in 38 studies; 3 studies used plasma; and one study used plasma exosomes. Furthermore, miRNA-205 and miRNA-9 were examined in three and two studies, respectively.

### Quality assessment

**Fig. 2:**
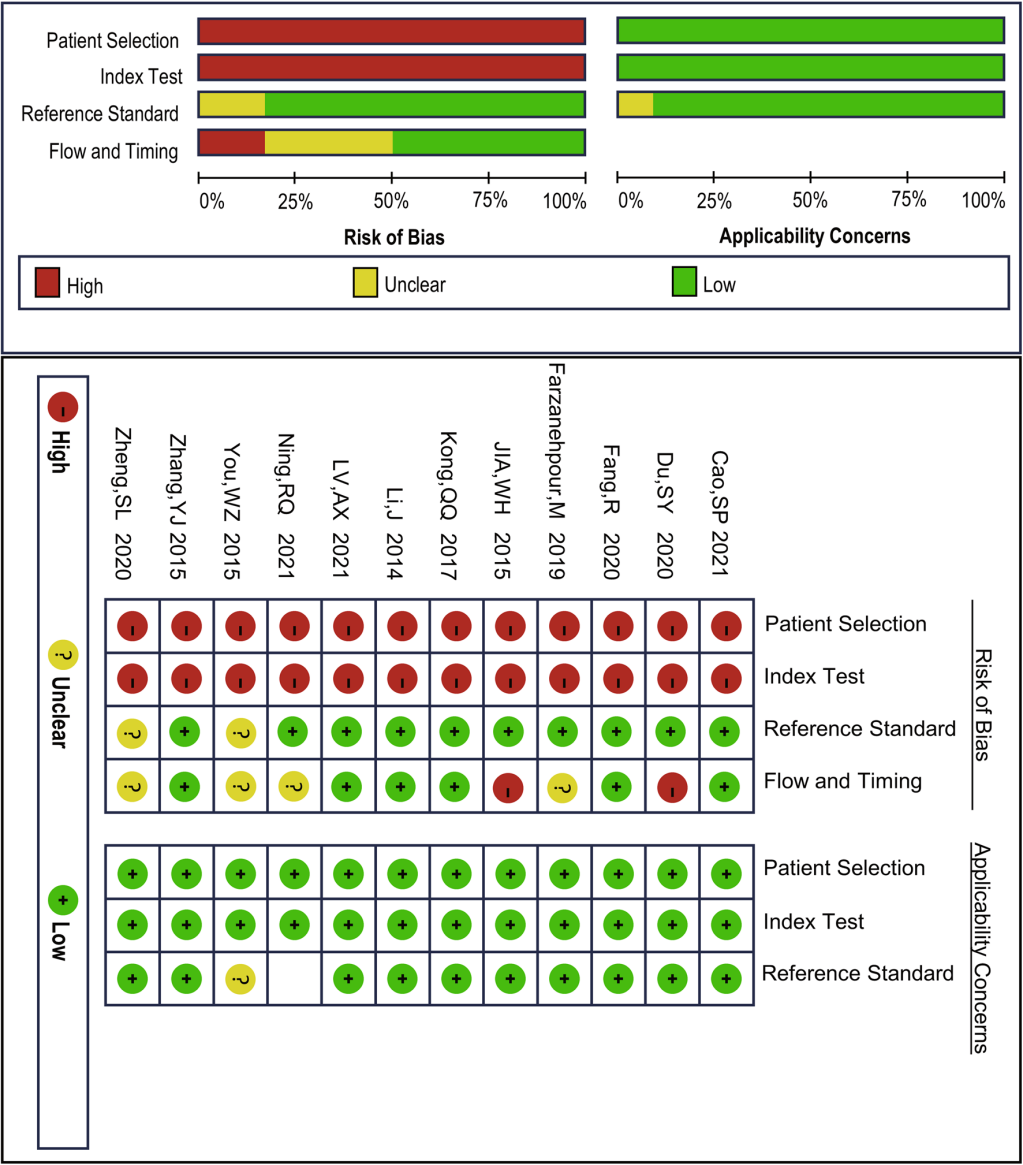
Quality Assessment of Diagnostic Accuracy Studies (QUADAS)-2 assessment for risk of bias and applicability. Red, yellow and green indicate high, unclear and low risk respectively

The QUADAS-2 was used to assess the quality of the 24 studies. As delineated in Fig. 2, every patient was diagnosed with a reference standard, resulting in minimal bias risk for patient diagnosis, as well as patient flow and timing. However, all studies were case‒control studies, leading to an increased risk of selection bias. Moreover, due to their retrospective nature, these studies collectively exhibit a considerable bias risk in the index test.

### Diagnostic accuracy of circulating miRNAs

**Fig. 3:**
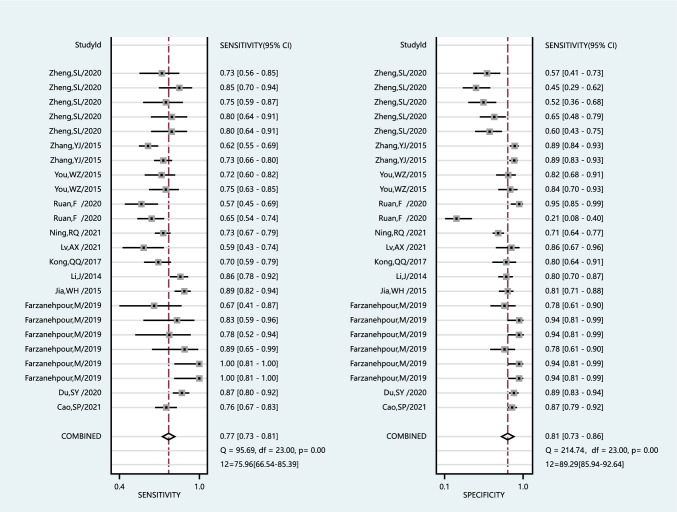
Forest plots show the sensitivity and specificity of miRNAs in the diagnosis of CIN and CC, respectively. The respective values and their confidence intervals

**Fig. 4:**
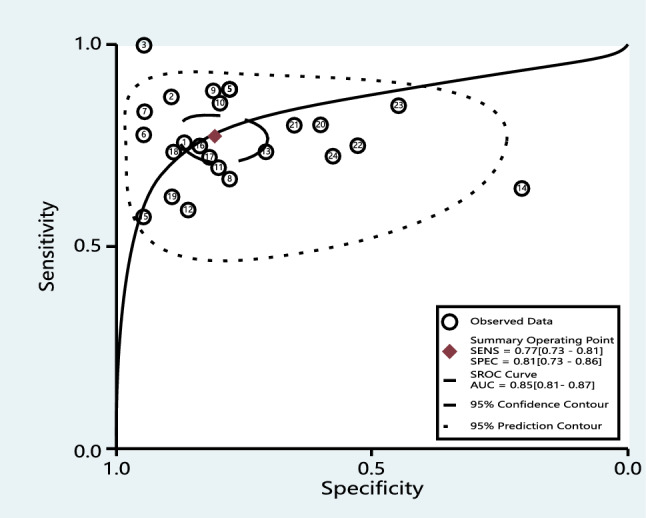
The sROC curve presents the overall accuracy of miRNAs in diagnosing CIN and CC

We chose the random effects model to examine the diagnostic performance of miRNAs for I^2^ > 50% (I^2^ = 75.96% for sensitivity and I^2^ = 89.29% for specificity, Fig. 3). The pooled diagnostic parameters for CIN and CC were as follows: the sensitivity was 0.77 (95% CI 0.73–0.81), the specificity was 0.81 (95% CI 0.73–0.86), the NLR was 0.28 (95% CI 0.23–0.35), the PLR was 3.99 (95% CI 2.81–5.65), and the DOR was 14.18 (95% CI 8.47–23.73). The AUC was 0.85 (95% CI 0.81–0.87) (Fig. 4). There was no evidence of a threshold effect (r = −0.093, P > 0.05).

**Fig. 5:**
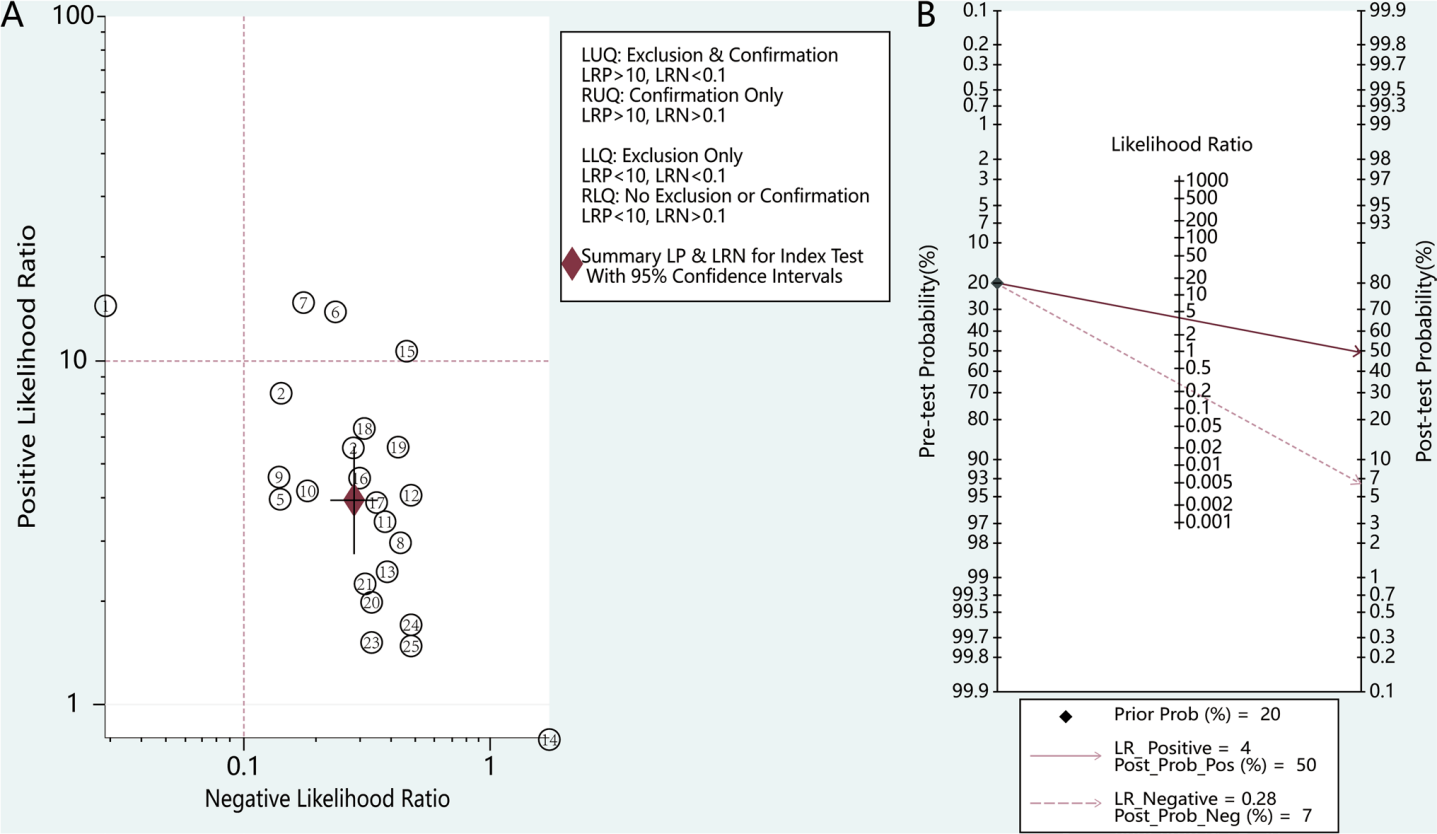
Assessment of clinical applicability of miRNAs in Diagnosing CIN and CC. **A**Summary of positive likelihood ratio and negative likelihood ratio for diagnosis of CIN and CC.; **B** Fagan’s nomogram evaluates the clinical utility of miRNAs for diagnosis of CIN and CC

The clinical applicability of the miRNAs was further evaluated by calculating both the PLR and NLR. High diagnostic accuracy was indicated by a PLR > 10 and NLR < 0.1. Notably, miR-9 and miR-192 showed potential as promising miRNAs for further investigation (Fig. 5A), and a study by Farzanehpour, M., et al. [14] Furthermore, Fagan’s nomogram corroborated the diagnostic accuracy of miRNAs, highlighting posttest probabilities of 50% and 7% for the PLR and NLR, respectively, when the pretest probability was set at 20% (Fig. 5B).

### Subgroup analyses and meta-regression of the diagnostic performance of miRNAs

**Fig. 6:**
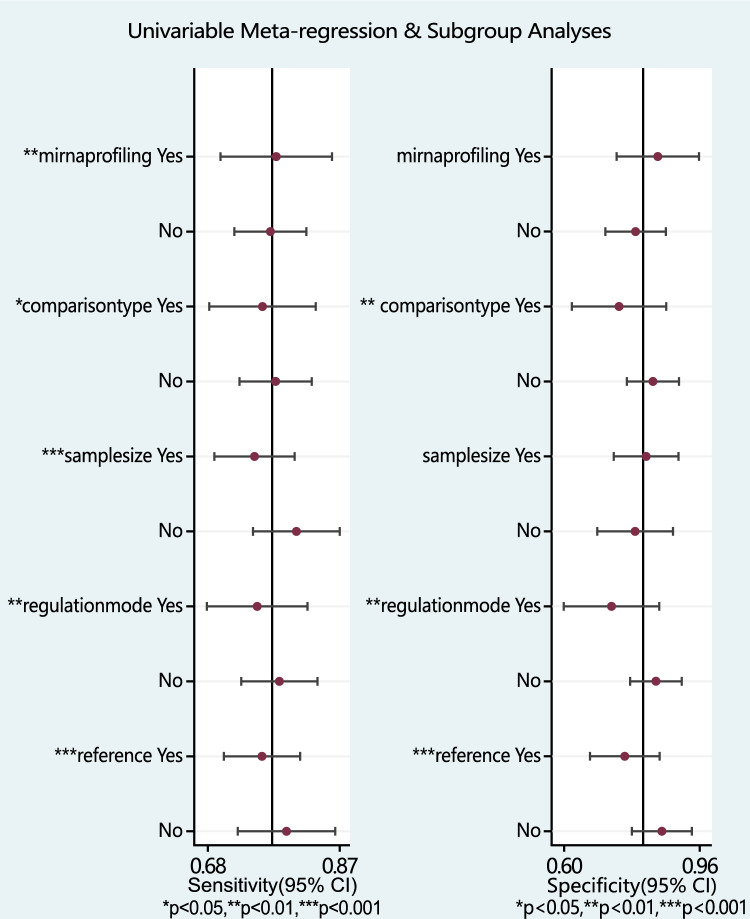
Univariable meta-regression and subgroup analyses for exploring the main sources of heterogeneity

To further investigate the heterogeneity, we conducted both subgroup (Table 3) and regression analyses (Fig. 6). Subgroup analyses revealed that multiple miRNA profiling and the U6 reference had a significant impact on diagnostic performance. The single miRNAs exhibited sensitivity, specificity, PLR, NLR, DOR, and AUC values of 0.77 (95% CI 0.71–0.82), 0.79 (95% CI 0.70–0.87), 3.73 (95% CI 2.43–5.73), 0.29 (95% CI 0.22–0.38), 12.82 (95% CI 6.80–24.17), and 0.83 (95% CI 0.80–0.86), respectively. Conversely, miRNA panels demonstrated sensitivity, specificity, PLR, NLR, DOR, and AUC values of 0.78 (95% CI 0.68–0.86), 0.85 (95% CI 0.78–0.90), 5.10 (95% CI 3.42–7.61), 0.26 (95% CI 0.17–0.39), 19.76 (95% CI 10.13–38.54), and 0.89 (95% CI 0.86–0.91), respectively.

**Table 3:** Summary estimates of diagnostic power and their 95% confidence intervals

Subgroup	No. studies	Sen (95% CI)	Spe (95% CI)	PLR (95% CI)	NLR (95% CI)	DOR (95% CI)	AUC (95% CI)	I^2^(%)
MiRNA profiling								
Single miRNA	19	0.77 (0.71–0.82)	0.79 (0.70–0.87)	3.73 (2.43–5.73)	0.29 (0.22–0.38)	12.82 (6.80–24.17)	0.83 (0.80–0.86)	78.8
Multiple miRNAs	5	0.78 (0.68–0.86)	0.85 (0.78–0.90)	5.10 (3.42–7.61)	0.26 (0.17–0.39)	19.76 (10.13–38.54)	0.89 (0.86–0.91)	88.2
Comparison type								
CC vs HC	13	0.80 (0.72–0.86)	0.85 (0.76–0.91)	5.24 (3.16–8.67)	0.23 (0.16–0.35)	22.36 (9.89–50.57)	0.89 (0.86–0.91)	84.5
CIN vs HC	9	0.74 (0.67–0.79)	0.74 (0.60–0.84)	2.80 (1.83–4.28)	0.36 (0.30–0.43)	7.79 (4.71–12.89)	0.78 (0.74–0.82)	63.6
Sample size								
< 100	12	0.82 (0.73–0.88)	0.79 (0.66–0.88)	3.94 (2.26–6.86)	0.23 (0.15–0.36)	17.07 (6.73–43.33)	0.87 (0.84–0.90)	68.7
≥ 100	12	0.75 (0.69–0.80)	0.82 (0.73–0.88)	4.16 (2.67–6.46)	0.31 (0.24–0.39)	13.59 (7.31–25.25)	0.84 (0.80–0.87)	86.9
Regulation* mode								
Down-regulate	9	0.76 (0.69–0.82)	0.74 (0.60–0.84)	2.89 (1.88–4.44)	0.32 (0.26–0.41)	8.97 (5.24–15.36)	0.81 (0.77–0.84)	67.7
Up-regulate	13	0.79 (0.72–0.85)	0.85 (0.77–0.90)	5.16 (3.18–8.36)	0.25 (0.18–0.34)	20.98 (9.80–44.89)	0.88 (0.85–0.90)	85.7
Reference								
U6	10	0.82 (0.70–0.89)	0.87 (0.74–0.94)	6.14 (2.84–13.27)	0.21 (0.12–0.37)	29.17 (8.76–97.08)	0.90 (0.87–0.93)	86.6
Non-U6	14	0.76 (0.71–0.81)	0.76 (0.68–0.83)	3.23 (2.37–4.39)	0.31 (0.25–0.38)	10.37 (6.68–16.11)	0.82 (0.79–0.85)	77.0

^*^Excluded research by Zhang, YJ. et al.[37] due to inconsistent miRNA expression changes

Moreover, among the selected studies, those using U6 as an internal reference showed a significant diagnostic value compared to the non-U6 group. The U6 subgroup had a sensitivity of 0.82 (95% CI 0.70–0.89), a specificity of 0.87 (95% CI 0.74–0.94), a PLR of 6.14 (95% CI 2.84–13.27), an NLR of 0.21 (95% CI 0.12–0.37), a DOR of 29.17 (95% CI 8.76–97.08), and an AUC of 0.90 (95% CI 0.87–0.93).

We also conducted meta-regression analyses, and the results showed that multiple miRNA profiles, using cervical cancer as the case group, a sample size less than 100, upregulated miRNAs and the use of U6 as a reference affected the sensitivity. Furthermore, using cervical cancer as the case group, miRNAs upregulation and using U6 as the reference affected the specificity.

### Sensitivity analyses

**Fig. 7:**
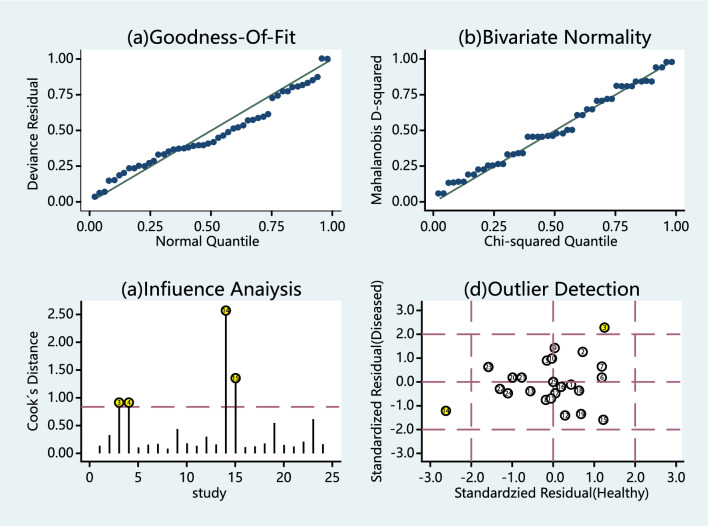
Diagram of sensitivity analysis showing (**a**) goodness-of-fit; **b** bivariate normality; **c** influence analysis; **d** outlier detection

The results of the sensitivity analysis are illustrated in Fig. 7, which shows that the appropriateness of the random effects model was validated based on the goodness of fit (Fig. 7A) and bivariate normality (Fig. 7B). The analysis of influence revealed that the studies by Farzanehpour, M. et al., and Ruan, F. et al. [15] exerted the greatest impact on the topic of weight (Fig. 7C). The identification of outliers in the data from the studies by Farzanehpour,M. et al., and Ruan,F. et al. (Fig. 7D) suggested that the observed heterogeneity may be attributed to these specific data points. Excluding three outlier groups reduced the degree of heterogeneity, as measured by the I^2^ value, by 3.93% for sensitivity and 3.72% for specificity. Despite excluding outlier groups, no statistically significant change was observed in the degree of heterogeneity (Table 4).

**Table 4:** Diagnostic performance of miRNAs in CIN and CC

Analysis	Overall	Outliers excluded
No. of studies	24	21
Sen (95% CI)	0.77 (0.73,0.81)	0.76 (0.72,0.80)
Spe (95% CI)	0.81 (0.73,0.86)	0.81 (0.74,0.86)
PLR (95% CI)	3.99 (2.81,5.65)	3.94 (2.98,5.20)
NLR (95% CI)	0.28 (0.23,0.35)	0.29 (0.25,0.35)
DOR (95% CI)	14.18 (8.47,23.73)	13.38 (9.27,19.32)
AUC (95% CI)	0.85 (0.81,0.87)	0.84 (0.81,0.87)

### Publication bias

**Fig. 8:**
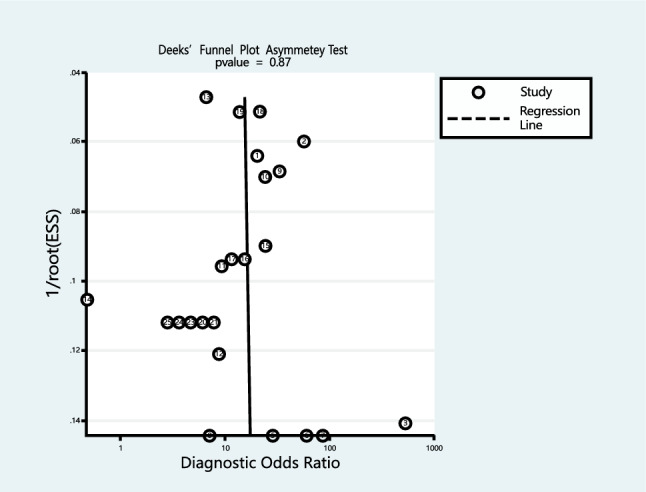
Deek’s funnel plot for evaluating potential publication bias

A funnel plot developed by Deek was employed to visualize the 24 studies included in the analysis. The generated plot, depicted in Fig. 8, exhibited a P-value of 0.87, suggesting no evidence of publication bias.

## Discussion

CC is one of the predominant gynaecological malignancies. Early detection of CIN and CC is an effective way to reduce the disease burden of cervical cancer. Recently, research has focused on circulating miRNAs as promising biomarkers for the early detection of CIN and CC [16, 17]. Our study aimed to evaluate the diagnostic performance of existing circulating miRNA-based screening methods discussed in the scholarly literature.

Our meta-analysis included 12 papers with 1817 CIN or CC patients and 1731 healthy controls. The results displayed a pooled sensitivity of 0.77 (95% CI 0.73–0.81) and specificity of 0.81 (95% CI 0.73–0.86). A PLR of 3.99 indicates that aberrant miRNA expression substantially increases the accuracy of detection of individuals with CIN and CC, similar to the pooled NLR. This analysis demonstrated a pooled DOR for miRNAs of 124.60, indicating their efficacy in differentiating patients (CC or CIN) from healthy individuals.

In our study, miR-9 and miR-192 showed potential as promising miRNAs for diagnosing CIN and CC. MiR-9 and miR-192 were significantly upregulated in the serum of cervical precancer patients [18]. ROC analysis revealed that miR-9 and miR-192 were highly accurate in discriminating CIN patients from healthy controls, with AUC values of 0.9 and 0.75, respectively [14].

Persistent infection with high-risk human papillomavirus (HPV) strains, particularly HPV-16, HPV-18, HPV-31, HPV-33, HPV-45, HPV-52, and HPV-58, significantly increases the risk of developing cervical lesions and their recurrence after treatment [19, 20]. Bogani's study found that patients with recurrent cervical lesions had longer HPV persistence than those without recurrence (12.2 vs. 9.0 months; p < 0.001) [21]. Additionally, the risk of lesion recurrence after primary conization is 7.46% at 6 months and rises to 13.1% at 12 months if HPV is still detected post-surgery. Moreover, adolescent HPV vaccination can decrease the prevalence of precancerous lesions and cervical cancer. HPV vaccination has been proved to prevent about 70% of lower genital tract dysplasia (LGTD) and reduce the risk of developing new infections of HPV. [22]. Bogani's study showed that vaccinations could offer up to a 67% protective effect [23].

The transformation of normal cervical cells infected with HPV into precancerous cells involves two main processes: the DNA damage response (DDR) and genome amplification of HPV. MiR-9 binds to the 3-UTR of FOXO3 and downregulates FOXO3 expression. FOXO3 inhibits DDR and induces p53-dependent apoptosis. Additionally, miR-9 could be associated with differentiation during the early transformation process. Epithelial-mesenchymal transition (EMT) converts epithelial cells into invasive and migratory mesenchymal cells [24]. The overexpression of miR-9 can lead to EMT, which is regulated by c-Myc and Prospero homeobox 1 (PROX1) [25, 26], contributing to cancer cell metastasis. MiR-192 promotes cell proliferation and migration and decreases apoptosis and cell cycle progression from G0/G1 to S phase by regulating important factors in this process [27, 28]. In colon cancer, it suppresses ZEB2 and VEGFA expression [29]. In renal and ovarian tumour models, it targets ZEB2 and RhoA and mediates TGF-beta-induced EMT [30]. However, its specific targets in cervical cancer remain unknown.

Owing to the heterogeneity of the included studies, we conducted subgroup and meta-regression analyses to examine covariates. Subgroup analysis identified miRNA profiling, comparison type, sample size, miRNA expression, and reference as potential sources of heterogeneity. The multiple miRNAs (AUC value of 0.89) showed better diagnostic accuracy than the single miRNAs (AUC value of 0.84). Our findings align with prior research by Du, SY. et al. [17]

In addition, our findings mandate the selection of suitable internal reference genes for standardization, preferring U6 over cel-39. qRT‒PCR is a common method for profiling circulating miRNA expression, and accurate interpretation depends heavily on selecting appropriate reference genes for normalization. However, the selection of appropriate internal reference genes is still debated [31]. Therefore, identifying consistent and highly stable internal reference genes is important for minimizing bias among tests.

Compared with previous studies [32], we performed subgroup analyses on miRNA regulation modes to investigate the origin of heterogeneity. We found that compared with downregulated miRNAs, upregulated miRNAs exhibited superior diagnostic performance. According to our meta-regression analysis, the regulation mode of miRNAs affects their sensitivity and specificity. Additionally, a similar article by Jiang et al. was published 3 years ago, whereas our study incorporates more recent studies exploring the use of miRNAs in the diagnosis of CC and CIN.

Nevertheless, this analysis has limitations. First, the lack of specified cut-off values for miRNAs in the included studies prevented us from performing a subgroup analysis, which might have resulted in heterogeneity. Second, as all studies included Asian populations, we could not investigate the impact of ethnicity. Third, we included only English language articles. Fourth, the predominance of retrospective case‒control studies increases the risk of selection bias. Last, due to the limited number of similar miRNAs available, it is currently not possible to identify a specific single miRNA or panel of miRNAs as the optimal diagnostic biomarkers for CIN and CC.

## Conclusion

In summary, current evidence suggests that circulating miRNAs are an encouraging noninvasive diagnostic tool for CIN and CC patients. Furthermore, miRNA panels possess superior diagnostic efficacy compared to that of individual miRNAs [33–38].

### Supplementary Information

Supplementary Material 1.

## Data Availability

Not applicable.
