# Supplementary material for: Circulating micrornas as potential diagnostic biomarkers for cervical intraepithelial neoplasia and cervical cancer: a systematic review and meta-analysis
Source: Discov Oncol. 2024 May 27;15:189. doi: 10.1007/s12672-024-01028-7 (PMC11130102; doi:10.1007/s12672-024-01028-7)
Supplement: Supplementary file 1 — Supplementary Material 1. [file 12672_2024_1028_MOESM1_ESM.docx]

Additional File 1 The complete search strategy of keywords

(((("Uterine Cervical Dysplasia"[Mesh]) OR ((((((((((((((((((((Cervical Dysplasia, Uterine[Title/Abstract]) OR (Dysplasia, Uterine Cervical[Title/Abstract])) OR (Dysplasia of Cervix Uteri[Title/Abstract])) OR (Cervix Uteri Dysplasia[Title/Abstract])) OR (Cervix Uteri Dysplasias[Title/Abstract])) OR (Cervical Intraepithelial Neoplasia[Title/Abstract])) OR (Cervical Intraepithelial Neoplasms[Title/Abstract])) OR (Cervical Intraepithelial Neoplasm[Title/Abstract])) OR (Intraepithelial Neoplasm, Cervical[Title/Abstract])) OR (Intraepithelial Neoplasms, Cervical[Title/Abstract])) OR (Neoplasm, Cervical Intraepithelial[Title/Abstract])) OR (Neoplasms, Cervical Intraepithelial[Title/Abstract])) OR (Intraepithelial Neoplasia, Cervical[Title/Abstract])) OR (Neoplasia, Cervical Intraepithelial[Title/Abstract])) OR (Cervical Dysplasia[Title/Abstract])) OR (Cervical Dysplasias[Title/Abstract])) OR (Dysplasia, Cervical[Title/Abstract])) OR (Cervix Dysplasia[Title/Abstract])) OR (Dysplasia, Cervix[Title/Abstract])) OR (Cervical Intraepithelial Neoplasia, Grade III[Title/Abstract]))) OR (("Uterine Cervical Neoplasms"[Mesh]) OR ((((((((((((((((((((((((((Cervical Neoplasm, Uterine[Title/Abstract]) OR (Cervical Neoplasms, Uterine[Title/Abstract])) OR (Neoplasm, Uterine Cervical[Title/Abstract])) OR (Neoplasms, Uterine Cervical[Title/Abstract])) OR (Uterine Cervical Neoplasm[Title/Abstract])) OR (Neoplasms, Cervical[Title/Abstract])) OR (Cervical Neoplasms[Title/Abstract])) OR (Cervical Neoplasm[Title/Abstract])) OR (Neoplasm, Cervical[Title/Abstract])) OR (Neoplasms, Cervix[Title/Abstract])) OR (Cervix Neoplasms[Title/Abstract])) OR (Cervix Neoplasm[Title/Abstract])) OR (Neoplasm, Cervix[Title/Abstract])) OR (Cancer of the Uterine Cervix[Title/Abstract])) OR (Cancer of the Cervix[Title/Abstract])) OR (Cervical Cancer[Title/Abstract])) OR (Uterine Cervical Cancer[Title/Abstract])) OR (Cancer, Uterine Cervical[Title/Abstract])) OR (Cancers, Uterine Cervical[Title/Abstract])) OR (Cervical Cancer, Uterine[Title/Abstract])) OR (Cervical Cancers, Uterine[Title/Abstract])) OR (Uterine Cervical Cancers[Title/Abstract])) OR (Cancer of Cervix[Title/Abstract])) OR (Cervix Cancer[Title/Abstract])) OR (Cancer, Cervix[Title/Abstract])) OR (Cancers, Cervix[Title/Abstract])))) AND (("MicroRNAs"[Mesh]) OR (((((((((((((((((MicroRNA[Title/Abstract]) OR (miRNAs[Title/Abstract])) OR (Micro RNA[Title/Abstract])) OR (RNA, Micro[Title/Abstract])) OR (miRNA[Title/Abstract])) OR (Primary MicroRNA[Title/Abstract])) OR (MicroRNA, Primary[Title/Abstract])) OR (Primary miRNA[Title/Abstract])) OR (miRNA, Primary[Title/Abstract])) OR (pri-miRNA[Title/Abstract])) OR (pri miRNA[Title/Abstract])) OR (RNA, Small Temporal[Title/Abstract])) OR (Temporal RNA, Small[Title/Abstract])) OR (stRNA[Title/Abstract])) OR (Small Temporal RNA[Title/Abstract])) OR (pre-miRNA[Title/Abstract])) OR (pre miRNA[Title/Abstract])))) AND (sensitiv*[Title/Abstract] OR sensitivity and specificity[MeSH Terms] OR (predictive[Title/Abstract] AND value*[Title/Abstract]) OR predictive value of tests[MeSH Term] OR accuracy*[Title/Abstract])
